# *Trypanosoma cruzi* infection associated with atypical clinical manifestation during the acute phase of the Chagas disease

**DOI:** 10.1186/s13071-019-3766-3

**Published:** 2019-10-30

**Authors:** Lucia Rangel-Gamboa, Lirio López-García, Francisco Moreno-Sánchez, Irma Hoyo-Ulloa, María Elisa Vega-Mémije, Nancy Mendoza-Bazán, Mirza Romero-Valdovinos, Angélica Olivo-Díaz, Guiehdani Villalobos, Fernando Martínez-Hernández

**Affiliations:** 1 Departamento de Ecología de Agentes Patógenos, Hospital General “Dr. Manuel Gea González”, Calzada de Tlalpan 4800, CP 14080 Mexico City, Mexico; 2 Private Practice, Dermatología clínica, Lomas Altas, Mexico City, Mexico; 3 Departamento de Infectología, Centro Médico ABC, Mexico City, Mexico; 4 Departamento de Dermatopatología, Hospital General “Dr. Manuel Gea González”, Calzada de Tlalpan 4800, CP 14080 Mexico City, Mexico; 5 Departamento de Genética e Histocompatibilidad, Hospital General “Dr. Manuel Gea González”, Calzada de Tlalpan 4800, CP 14080 Mexico City, Mexico

**Keywords:** Cutaneous disseminated infection, *Trypanosoma cruzi*, Acute phase of the Chagas disease

## Abstract

**Background:**

Chagas disease (CD) is caused by the protozoan parasite *Trypanosoma cruzi* and is transmitted by triatomine insects. Clinical manifestations vary according to the phase of the disease. Cutaneous manifestations are usually observed in the acute phase (chagoma and Romaña’s sign) or after reactivation of the chronic phase by immunosuppression; however, a disseminated infection in the acute phase without immunosuppression has not been reported for CD. Here, we report an unusual case of disseminated cutaneous infection during the acute phase of CD in a Mexican woman.

**Methods:**

Evaluation of the patient included a complete clinical history, a physical exam, and an exhaustive evaluation by laboratory tests, including ELISA, Western blot and PCR.

**Results:**

Skin biopsies of a 50-year-old female revealed intracellular parasites affecting the lower extremities with lymphangitic spread in both legs. The PCR tests evaluated biopsy samples obtained from the lesions and blood samples, which showed a positive diagnosis for *T. cruzi.* Partial sequencing of the small subunit ribosomal DNA correlated with the genetic variant DTU II; however, serological tests were negative.

**Conclusions:**

We present a case of CD with disseminated skin lesions that was detected by PCR and showed negative serological results. In Mexico, an endemic CD area, there are no records of this type of manifestation, which demonstrates the ability of the parasite to initiate and maintain infections in atypical tissues
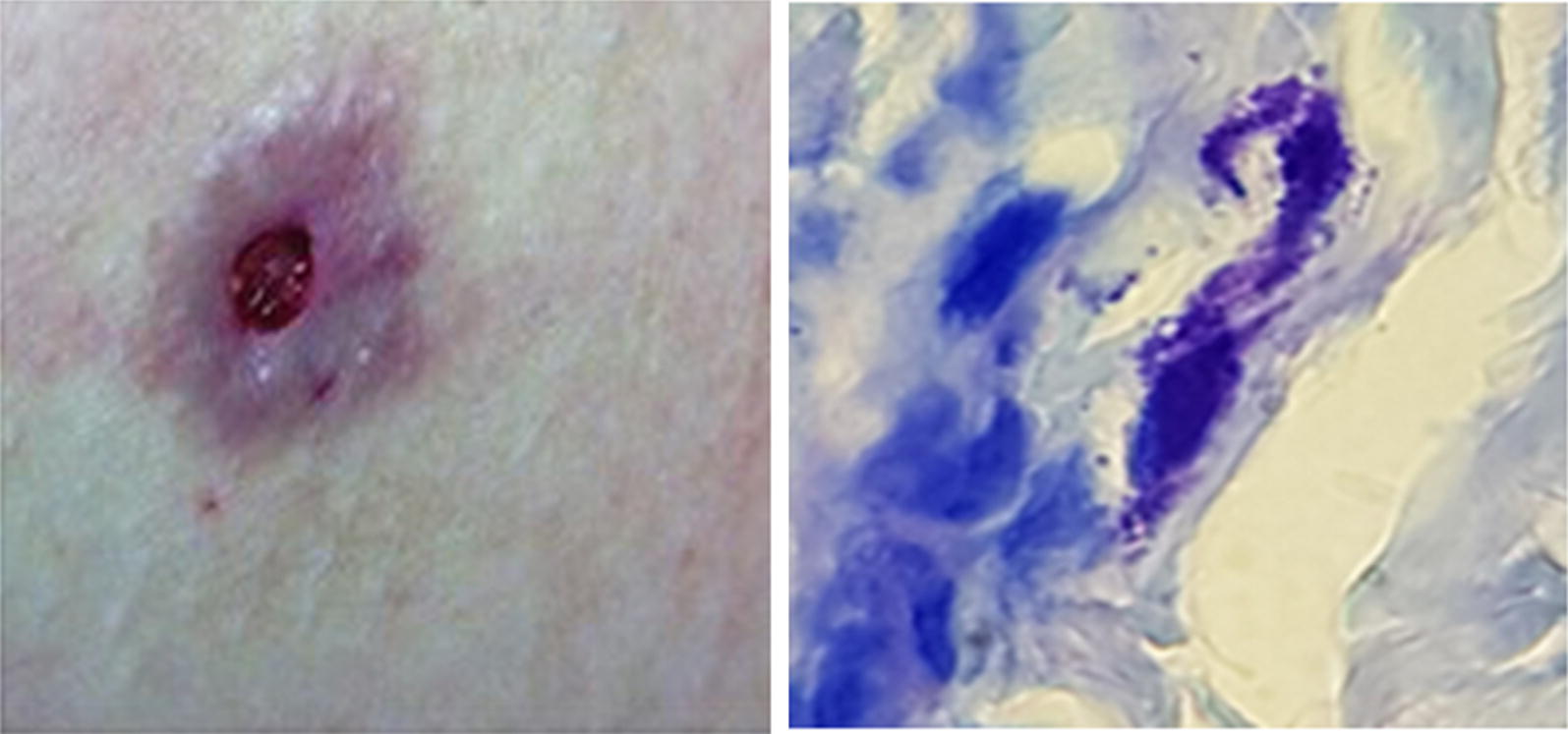
.

## Background

CD is an anthropozoonosis caused by the protozoan parasite *Trypanosoma cruzi* and is transmitted by various species of blood-sucking triatomine insects (kissing bugs). *Trypanosoma cruzi* originates in the Americas, where it is considered a major social and public health problem [1, 2]. CD has become a concern in the developed world because of human migration; thus, physicians worldwide are familiar with this disease [3, 4], and global warming and other factors further increase vector distribution [5].

CD has three phases: acute, indeterminate and chronic. The acute phase occurs immediately following infection, and only 5% of individuals show symptoms during this phase. Symptoms during this phase can include malaise and fever, which may conclude between four and eight weeks later. Cutaneous manifestations are frequent during the acute phase and may include localized inflammation at the site of inoculation (chagoma), unilateral palpebral edema (Romaña’s sign) and a generalized morbilliform eruption (schizotrypanides). In this phase, the presence of parasites in the blood is observed, which makes diagnosis by PCR highly sensitive, whereas serological tests are not conclusive [1, 2, 6].

Individuals in the indeterminate phase are asymptomatic, and 20–40% of infected individuals will progress to the chronic phase, which is characterized by cardiovascular (heart failure, arrhythmia and thromboembolism) or digestive (megacolon and megaesophagus) complications [1–6]. Cutaneous manifestations in the chronic phase are extremely rare and have been observed only as a result of reactivation of the infection in immunocompromised individuals (HIV/AIDS) or in infected individuals undergoing immunosuppressive treatment for an organ transplant [7–15]. This reactivation is characterized by the presence of amastigotes in skin biopsies, fever and positive serological tests for *T. cruzi* infection. However, a disseminated infection has not been observed in the acute or chronic phase of the disease to date.

In this report, we present an unusual case of cutaneous disseminated CD in Mexico, highlighting that this type of parasite reaction is extremely rare.

## Methods

### Serological diagnosis

ELISAs and Western blots were performed to determine the presence of antibodies against *T. cruzi*. In both cases, the total protein extract of the Ninoa strain (MHOM/MX/1994/Ninoa) was used as the antigen. Both tests were performed as indicated by Rangel-Flores et al. [16]. Control sera that were positive and negative for *T. cruzi* infection were evaluated previously in a routine checkup by the blood bank at the General Hospital Dr Manuel Gea Gonzalez. For the ELISA, each sample was evaluated in triplicate, and the cut-off point (CO) was calculated according to the equation CO = m + 2.5δ, where m is the average absorbance of the negative samples and δ is the standard deviation.

### DNA isolation and PCR

DNA was extracted from biopsy samples that were embedded in paraffin. Briefly, the samples were dewaxed with 100% xylol and incubated at 55 °C for 30 min and then centrifuged at 18,800×*g* for 5 min. The supernatant was then removed, and the samples were hydrated in 1 ml sequential steps with ethanol (100, 90, 80 and 70%) [17]. The samples were then placed in 1 ml of lysis solution (50 mM Tris-HCl; 50 mM EDTA, pH 8; 50 mM NaCl; 1% SDS and 20 μg/ml proteinase K), macerated with a homogenizer (Pro Scientific, pro200, Oxford, USA) and incubated at 55 °C overnight. The phenol-chloroform technique was used to extract DNA [18].

Universal primers designed in the laboratory were used to amplify a region of conserved sequences of small subunit ribosomal DNA (*SSU* rDNA) of the family Trypanosomatidae; the forward primer Trypanosomatidae18SF 5ʹ-ATC TGG TAA AGT TCC CCG TG-3ʹ and the reverse primer Trypanosomatidae18SR 5ʹ-CCG TTT CGG CTT TTG TTG GT-3ʹ were used to amplify a fragment of 830 bp. In addition, a diagnostic PCR assay using a DNA satellite of *T. cruzi* was performed using the primers TCZ_F (5ʹ-GCT CTT GCC CAC AMG GGT GC-3ʹ) and TCZ_R (5ʹ-CCA AGC AGC GGA TAG TTC AGG-3ʹ), which amplify a region of 188 bp; the satellite DNA PCR conditions were set according to Elias et al. [19]. To discard the presence of other related parasites, such as *Leishmania*, the following primers were used: LM9 (5ʹ-GGA CGA GCT CAT GGC GCC-3ʹ) and LM12 (5ʹ-CTG GCA CAC CTC CAC GTA C-3ʹ), and for the *L. mexicana* complex, IR1 (5ʹ-GCT GTA GGT GAA CCT GCA GCA GCT GGA TCA TT-3ʹ) and LM17 (5ʹ-CCC CTC TCC TCC TCC CC-3ʹ); the PCR conditions were performed according to Berzunza et al. [20].

PCR amplifications for small subunit ribosomal DNA (*SSU* rDNA) were carried out in a final volume of 25 μl, containing 10 pmol of each primer, 1× PCR buffer (8 mM Tris-HCl, pH 8; 20 mM KCl), 1.5 mM MgCl_2_, 0.5 mM dNTPs, and 2 U of Taq DNA Polymerase (Promega, Madison, WI, USA). Approximately 500 to 1000 ng of DNA was used as a template to amplify genomic sequences. The amplification conditions were 1 cycle at 94 °C for 5 min, 35 cycles including denaturation, annealing and extension steps at 94 °C for 30 s, 54 °C for 1 min and 72 °C for 30 s, respectively, and a final extension step at 72 °C for 7 min. The presence of amplicons was observed by electrophoresis in a 1.5% agarose gel, after which the band was purified using an AxyPrep PCR clean-up kit (Axigen Biosciences, Union City, CA, USA) and sequenced on both strands by a commercial supplier (Instituto de Biologia, Universidad Nacional Autonoma de Mexico).

### Phylogenetic reconstruction and network tree

The sequences obtained in this study were subjected to a BLAST search in the GenBank database and were submitted with the number MK640442. Multiple alignments were performed using the CLUSTAL W and Muscle programs and manually adjusted with MEGA 5.05 software [21]. The best-fit model of nucleotide substitution was the Hasegawa-Kishino-Yano model with gamma distribution and invariable sites, as determined using the Akaike information criterion in Modeltest software, version 3.7 [22]. Phylogenetic reconstruction was performed using Mr Bayes 3.1.2 software [23]. The analysis was performed over 10 million generations, with trees sampled every 100 generations. Trees with scores lower than those at the stationary phase (‛burn-inʼ) were discarded. The trees that reached the stationary phase were collected and used to build consensus trees. Other sequences were collected from GenBank and used as DTU references and for population genetics analysis (Additional file 1: Table S1). Median-joining network [24] was constructed using NETWORK 4.611 (http://fluxusengineering.com) with the default settings and assumptions.

## Results

**Fig. 1:**
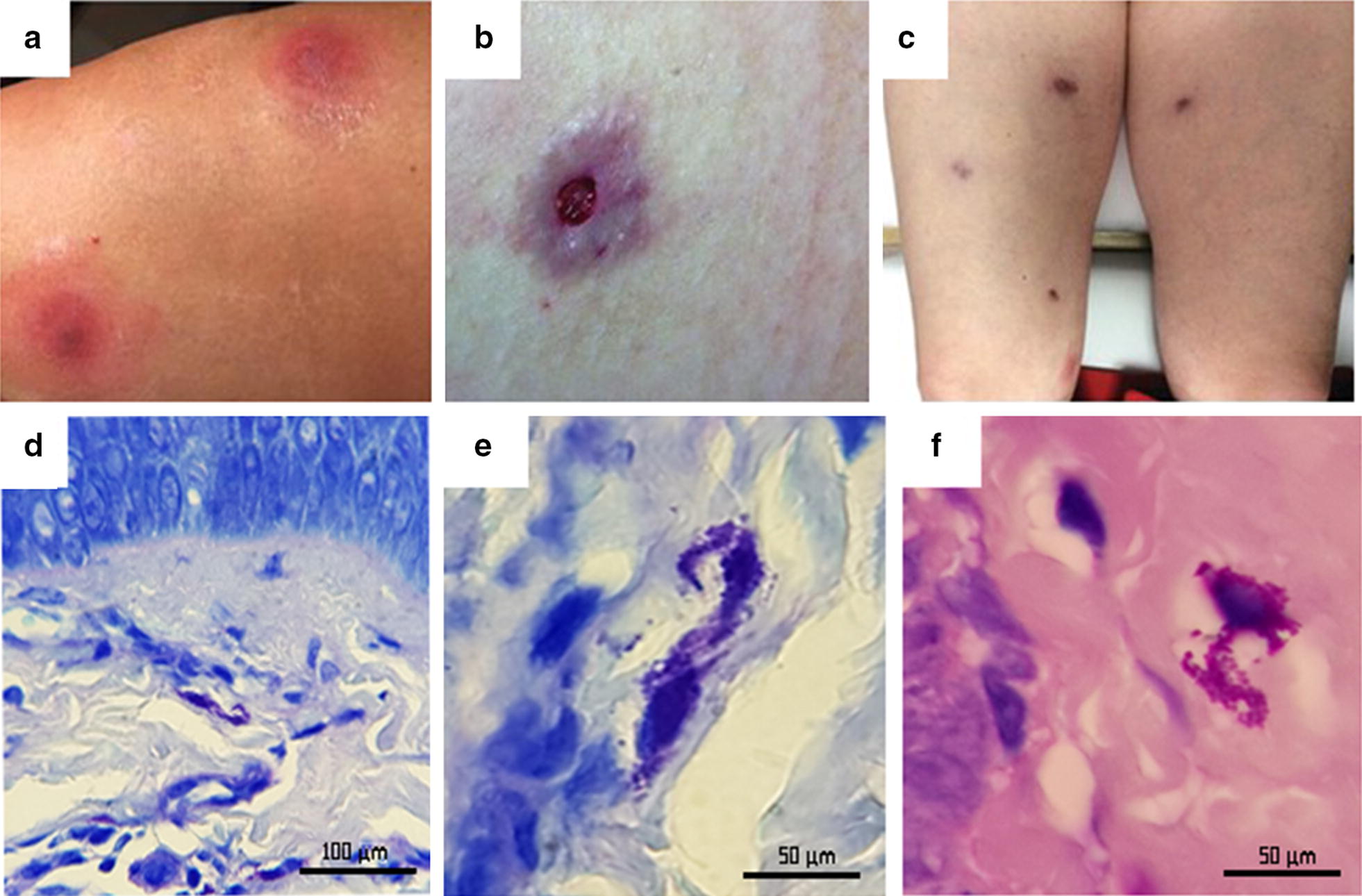
Subcutaneous nodules (**a**), ulcer (**b**), scars and subcutaneous nodules in both legs (**c**). **d** Photomicrograph of deep dermis with inflammatory infiltrate and infected cell (Giemsa staining; 400×). **e** Photomicrograph showing intracellular amastigote nests (Giemsa staining; 1000×). **f** Photomicrograph showing intracellular amastigote nests (H&E straining; 1000×). *Scale-bars*: **d**, 100 µm; **e**, **f**, 50 µm

A 50-year-old woman from Mexico City presented with cutaneous non-healing ulcers affecting the lower extremities, which had been treated with multiple antibiotics. The present illness initiated with erythematous subcutaneous nodules that suddenly appeared in both thighs. Over one week, the nodules increased in size and developed central necrosis, after which some of them ulcerated (Fig. 1a, b). The patient had stayed in Baja California, Mexico, where she received multiple bites from different insects a few days before the initiation of the current dermatosis.

The relevant medical background includes cholecystectomy, hysterectomy, hypothyroidism, systemic arterial hypertension and dyslipidemia treated with liothyronine/levothyroxine, olmesartan/hydrochlorothiazide and atorvastatin, respectively. A diagnosis of viral myocarditis was made the previous year after, with negative results for other parasites, including *T. cruzi* infection.

Physical examination revealed diffuse subcutaneous nodules with lymphangitic spread in both legs (Fig. 1c); nevertheless, the vital signs, complete blood count (CBC) and blood chemistry remained within the normal parameters.

Additionally, two skin biopsies were obtained, one from a subcutaneous nodule and the other from an ulcerated lesion. Histopathological analysis revealed an epidermis with irregular acanthosis, a deep dermis and adipose tissue with dense inflammatory infiltrate (Fig. 1d); macrophages were observed whose cytoplasm contained numerous round or ovoid microorganisms with a central nucleus and kinetoplast, which were morphologically compatible with amastigotes (Fig. 1e, f).

**Fig. 2:**
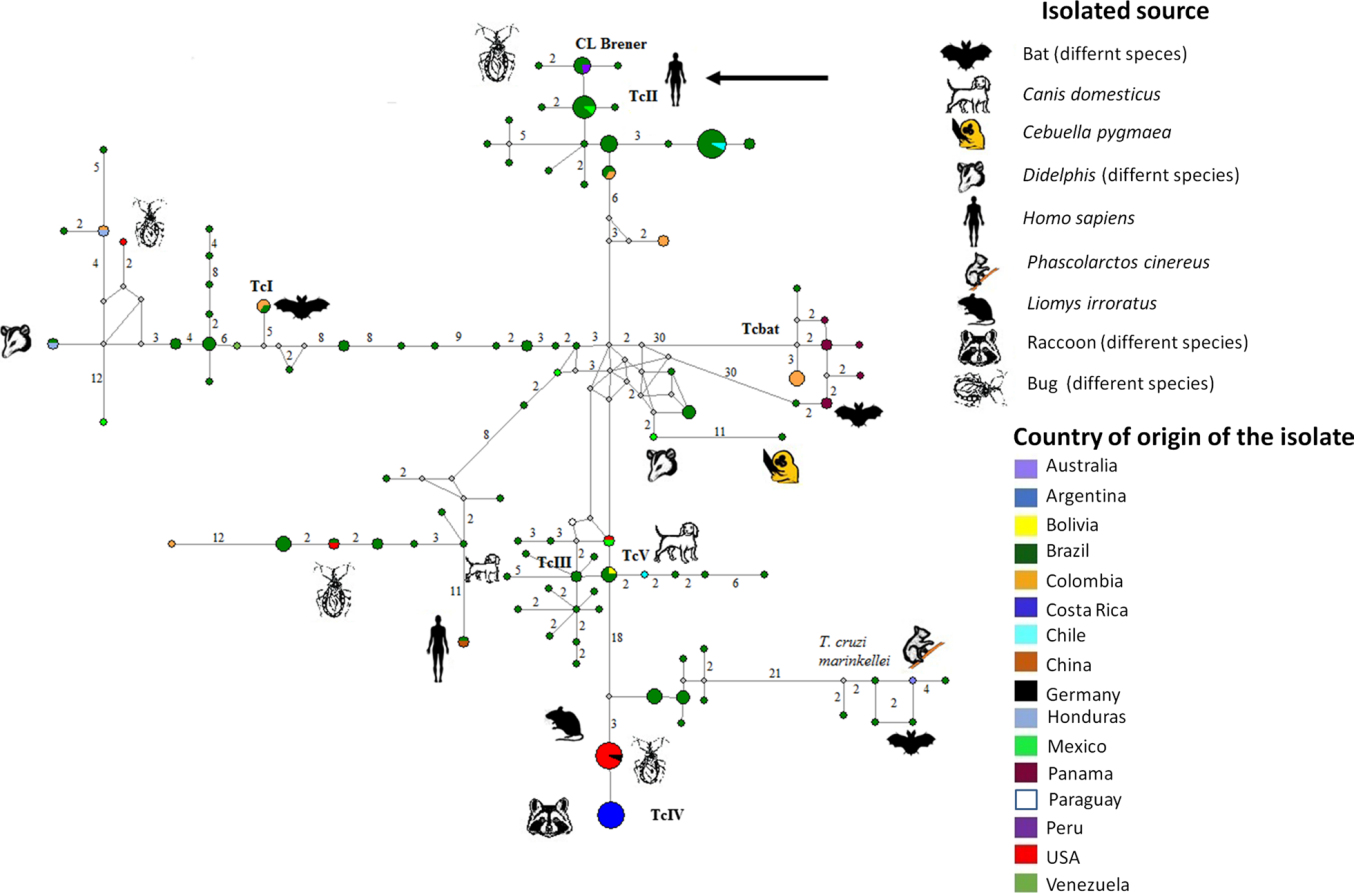
Haplotype network constructed with *SSU* ribosomal DNA sequences from different *Trypanosoma cruzi* DTUs sequences available on GenBank. The numbers refer to mutational changes; the sizes of circles are proportional to haplotype frequencies and the color indicates the geographical distribution; the black arrow indicates the sequences obtained in the present study (GenBank: MK640442)

Considering the histopathological results and the dissemination of the cutaneous lesions, a leishmaniasis diagnosis was suggested; therefore, treatment with itraconazole together with an antimonial for local infiltration was started. Despite some initial clinical improvement, the lesions evolved to erythematous fibrous scars. Approximately 12 weeks later, a group of new disseminated lesions appeared (Fig. 1c), which resembled the original nodular lesions and displayed lymphangitic spread. At this time, bacterial, mycobacterial and fungal cultures, as well as, *Leishmania* evaluation by PCR tests were negative. Thus, a PCR was performed to determine the presence of Trypanosomatidae with DNA isolated from the biopsies and blood; with a positive result, and the partial *SSU* rDNA sequences obtained showed an identity of 99% with the *T. cruzi* Y30 clone (GenBank: JN942610). Bayesian phylogenetic analysis (Additional file 2: Figure S1) showed high posterior probability values (1.00) for DTU II and DTU VI; however, the network trees (Fig. 2) showed a clear association with the DTU II of *T. cruzi*, while satellite DNA sequences (Additional file 3: Text S1) showed a 98% (180/183) of identity to *T. cruzi* isolates from southern Texas (GenBank: LT220305).

A complete cardiovascular evaluation (electrocardiogram, transthoracic echocardiogram and barium swallow) was performed with normal results; thereafter, cardiovascular follow-up was recommended. The serological ELISA and WB tests were negative for the presence of antibodies against *T. cruzi*; the ELISA value was 0.12 of absorbance, below the CO point (0.18), and no specific antigens were detected in the WB test (data not shown).

## Discussion

Here, we present a case of CD characterized by subcutaneously disseminated skin lesions that displayed lymphangitic spread in both legs; the lesions appeared two weeks after a trip to Baja California, Mexico. In this region of Mexico, the presence of six species of triatomine insects has been reported, and serological tests have indicated a high prevalence of *T. cruzi* infection [25, 26]. Based on the patient’s description, we hypothesized that the transmission route was vectorial and that the infection was acquired in northern Mexico; nevertheless, we do not dismiss other possible routes and areas of infection.

The clinical diagnosis was erythema nodosum, which is classified as a form of panniculitis (subcutaneous hypersensitivity reaction). Different forms of panniculitis have been reported during the reactivation of CD in the chronic phase [7–15], in which the presence of parasites was observed in the lesions but not in the blood. In this study, the diagnosis of *T. cruzi* was made in samples obtained from both blood and histopathological skin examination, with negative serological tests, so we suggest an acute infection. To the best of our knowledge, this is the first reported case of a disseminated cutaneous infection in the acute phase, at least among the cases registered in Mexico.

Due to the disseminated skin lesions, different diagnoses were considered, among them the presence of *Leishmania* spp. infections; however, the PCR analyses did not show specific amplifications for any *Leishmania* species, including the *L. mexicana* complex, suggesting that the disseminated skin lesions were produced exclusively by *T. cruzi*. Interestingly, the *T. cruzi SSU* rDNA sequence obtained in this work corresponds to DTU II, which is also a novel finding, considering that the genotype circulating in humans, reservoirs and vectors in Mexico is predominantly DTU I [27, 28]. The presence of DTU II has not been reported in northern Mexico, and there is only one report in southern Mexico corresponding to infections in reservoirs [29]. The satellite DNA sequences showed 98% of identity to isolates from southern Texas, which were associated with rodents, and the high identity suggests that similar genetic variants of these parasites are circulating between Mexico and the USA. This last finding agrees with the hypothesis that the patient was infected in northern Mexico.

Regarding the patient’s medication intake before infection, some reports have evaluated the relationship between the medications and *T. cruzi* infection. Zhao et al. [30] observed that in murine models, atorvastatin resulted in the overexpression of low-density lipoprotein (LDL) receptors, enhancing the number of parasites in tissues, inflammation and heart tissue damage, associated with high mortality rates during acute *T. cruzi* infection. Furthermore, Nagajyothi et al. [31] observed that LDL receptors play an important role in the internalization of the parasite. On the other hand, olmesartan decreases IL-1β and TNF-α and increases IL-10 [32], which could help the parasite establish an infection by decreasing the levels of proinflammatory cytokines [33]. Liothyronine has been associated with decreased immunoglobulins in the plasma of birds, but this effect is unknown in humans [34]. The combination of atorvastatin, liothyronine and olmesartan may have created a unique environment for *T. cruzi*, suggesting that perturbation of the immune system rather than immune suppression may affect the clinical presentation and infection development. Further studies should be conducted to determine the real impact of these drugs on *T. cruzi* infection and whether the administration of these drugs can induce a disseminated infection.

Finally, this type of report is important because characterization of this disease is difficult due to its similarity to other diseases; likewise, skin lesions caused by *T. cruzi* are scarcely reported in the literature, probably because the parasite is associated with tropism to other tissue types. However, these findings demonstrate the ability of the parasite to infect and maintain atypical lesions.

## Conclusions

We present a case of disseminated skin lesions that were induced by *T. cruzi*, characterized by the presence of parasites in both the blood and lesions as detected by PCR, with negative serological diagnoses. This is the first case reported of a disseminated cutaneous infection in the acute phase without apparent immunosuppression in the patient.

## Supplementary information

**Additional file 1: Table S1.** Information for the sequences available on GenBank used to construct the haplotype network.

**Additional file 2: Figure S1.** Phylogenetic reconstruction using *SSU* rDNA for different trypanosomatid species available on GenBank.

**Additional file 3: Text S1.** Sequence obtained from the patient for the satelite-DNA region of *Trypanosma cruzi.*

## Data Availability

Data supporting the conclusions of this article are included within the article and its additional files. The datasets used and/or analyzed during the present study are available from the corresponding author upon request.

## Abbreviations

CD: Chagas disease
*SSU* rDNA: *18S* small subunit ribosomal DNA
PCR: polymerase chain reaction
DTU: discrete typing units
DTUI: discrete typing unit I
DTUII: discrete typing unit II
