# Supplementary material for: Trypanosoma cruzi infection associated with atypical clinical manifestation during the acute phase of the Chagas disease
Source: Parasit Vectors. 2019 Oct 30;12:506. doi: 10.1186/s13071-019-3766-3 (PMC6822409; doi:10.1186/s13071-019-3766-3)
Supplement: Supplementary file 1 — Additional file 1: Table S1. Information for the sequences available on GenBank used to construct the haplotype network. [file 13071_2019_3766_MOESM1_ESM.doc]

**Additional file 1:** **Table S1.** Information for the sequences available on GenBank used to construct the haplotype network.

| **Ascension number** | **Species** | **Country of isolation** | **Isolate or strain** | **Reported DTU** | **Host** | **References** |
| --- | --- | --- | --- | --- | --- | --- |
| AF228685 | *Trypanosoma cruzi* | Brazil | NRCl3 | TcV | *Homo sapiens* | [[1]](#endnote-2), [[2]](#endnote-3) |
| AF232214 | *T. cruzi* | Bolivia | Sc43Cl1 | TcIId, or TcV/VI | *Homo sapiens* | 1, [[3]](#endnote-4) |
| AF239980 | *T. cruzi* | Colombia | Colombiana | TcI | *Homo sapiens* | 1 |
| AF239981 | *T. cruzi* | Brazil | G | TcI | *Didelphis marsupialis* | 1 |
| AF245380 | *T. cruzi* | Brazil | Yuyu |  | *Bug* | 1 |
| AF245382 | *T. cruzi* | Venezuela | Dm28c | TcI | *Didelphis sp.* | 1,3 |
| AF245381 | *T. cruzi* | Argentina | CA-1 |  | *Homo sapiens* | 1 |
| AF245383 | *T. cruzi* | Brazil | CL Brener | Tc III | *Triatoma infestans* | 1 |
| AF288660 | *T. cruzi* | Brazil | MT3663 | TcIII/V | *?* | 1, 3 |
| AF288661 | *T. cruzi* | Brazil | MT4167 | TcI |  | 1 |
| AF292942 | *T. cruzi* | Brazil | MT4166 |  |  | 1 |
| AF301912 | *T. cruzi* | Brazil | Y | TcII | *Homo sapiens* | 1, 3 |
| AF303659 | *T. cruzi* | Brazil | Silvio X10 cl1 | TcI | *Homo sapiens* | 1, 3 |
| AF303660 | *T. cruzi* | Brazil | MT3869 | TcIII | *Homo sapiens* | 1, 3 |
| AF359461 | *T. cruzi* | Brazil | IevolMT3663 |  | Biochemical Experimental | [[4]](#endnote-5) |
| AF359462 | *T. cruzi* | Brazil | IevolMT3663 |  | Biochemical Experimental | 4 |
| AF359463 | *T. cruzi* | Brazil | IevolMT3663 |  | Biochemical Experimental | 4 |
| AF359464 | *T. cruzi* | Brazil | IevolMT3663 |  | Biochemical Experimental | 4 |
| AF359465 | *T. cruzi* | Brazil | IevolMT3663 |  | Biochemical Experimental | 4 |
| AF359466 | *T. cruzi* | Brazil | IevolMT3663 |  | Biochemical Experimental | 4 |
| AF359467 | *T. cruzi* | Brazil | IevolMT3663 |  | Biochemical Experimental | 4 |
| AF359468 | *T. cruzi* | Brazil | IevolMT3663 | TcIII | Biochemical Experimental | 4 |
| AJ009147 | *T. cruzi* | Brazil | Sylvio X10 | TcI | *Homo sapiens* | [[5]](#endnote-6), [[6]](#endnote-7) |
| AJ009148 | *T. cruzi* | Brazil | CAN III (clone 1) | Tc IV | *Homo sapiens* | 2, 3, 5, 6 |
| AJ009149 | *T. cruzi* | Chile | VINCH 89 | TcV | *Triatomine infestans* | 5, 6 |
| AJ009150 | *T. cruzi marinkellei* | Brazil | B7 | *marinkellei* | *Phyllostomum discolor* (Bat) | 5, 6 |
| AJ620544 | *T. cruzi* | Germany | monkey Germany |  | *Macaca mulatta* | 6 |
| AY491761 | *T. cruzi* | Brazil | Jose Julio | TcIV |  | 3, [[7]](#endnote-8) |
| AY491762 | *T. cruzi* | Brazil | 338 | TcIIa |  | 7 |
| AY491763 | *T. cruzi* | Brazil | 262/AEAAB | TcI | *Cebuella pygmaea* | 7 |
| AY785561 | *T. cruzi* | Brazil | Basileu | TcII | ? | Unpublished |
| AY785563 | *T. cruzi* | Brazil? | Esmeraldo cl3, clone 1 | TcII/VI | ? | 2, 3 |
| AY785564 | *T. cruzi* | Brazil | Esmeraldo cl3,clone 2 | TcII/VI | ? | 3 |
| AY785565 | *T. cruzi* | Brazil | Tc3014, clone 1 |  | ? | Unpublished |
| AY785566 | *T. cruzi* | Brazil | Tc3014, clone 2 |  | ? | Unpublished |
| AY785567 | *T. cruzi* | Brazil | TcXEN35, clone 1 | TcII | ? | Unpublished |
| AY785568 | *T. cruzi* | Brazil | TcXEN35, clone 2 |  | ? | Unpublished |
| AY785569 | *T. cruzi* | Brazil | Hem 179, clone 1 | TcVI | ? | Unpublished |
| AY785570 | *T. cruzi* | Brazil | Hem 179, clone 2 |  | ? | Unpublished |
| AY785572 | *T. cruzi* | Brazil | TcP06 | TcI | ? | Unpublished |
| AY785573 | *T. cruzi* | Brazil | Jose, clone 1 |  | ? | Unpublished |
| AY785574 | *T. cruzi* | Brazil | Jose, clone 2 |  | ? | Unpublished |
| AY785575 | *T. cruzi* | Brazil | SLU142, clone 1 |  | ? | Unpublished |
| AY785577 | *T. cruzi* | Brazil | M6241 cl6, clone 1 |  | ? | Unpublished |
| AY785578 | *T. cruzi* | Brazil | M6241 cl6, clone 2 | TcIII/V | ? | 3 |
| AY785579 | *T. cruzi* | Brazil | SO3 cl5, clone 1 | TcIII/TcV | ? | 3 |
| AY785580 | *T. cruzi* | Brazil | SO3 cl5, clone 2 | TcIII/TcV | ? | 3 |
| AY785581 | *T. cruzi* | Honduras | Honduras, clone 1 |  | ? | Unpublished |
| AY785582 | *T. cruzi* | Honduras | Honduras, clone 2 |  | ? | Unpublished |
| AY785583 | *T. cruzi* | Brazil | Famema | TcII | ? | Unpublished |
| CP015657 | *T. cruzi cruzi* | Brazil | Sylvio X10/cl1, clone 7 | TcI | *Homo sapiens* (blood) | [[8]](#endnote-9), [[9]](#endnote-10) |
| CP015675 | *T. cruzi cruzi* | Brazil | Sylvio X10/cl1 | TcI | *Homo sapiens* (blood) | 8, 9 |
| EU755228 | *T. cruzi* | brazil | TryCC 698 | TcIIa | *Rhodnius robustus* | [[10]](#endnote-11) |
| EU867806 | *T. cruzi* | Brazil | TryCC 1109 | TcI | *Rhodnius stali*(Triatomine) | [[11]](#endnote-12) |
| EU867807 | *T. cruzi* | Brazil | TryCC 1116 | TcI | *Rhodnius stali*(Triatomine) | 10 |
| EU867809 | *T. cruzi marinkellei* | Brazil | TryCC 1093 | marinkellei | *Artibeus planirostris* | 10 |
| FJ001616 | *T. cruzi* | Brazil | TCC/USP:139 | TcII | *Didelphis aurita* | 2, [[12]](#endnote-13) |
| FJ001619 | *T. cruzi* | Brazil | TCC/USP:294 | TcBat | *Myotis levis* | 12 |
| FJ001621 | *T. cruzi* | Brazil | TCC/USP:335 | TcII or TcIIb | *Homo sapiens* | 12 |
| FJ001624 | *T. cruzi* | Brazil | TCC/USP:642 | TcI | *Carollia perspicillata* | 12 |
| FJ001625 | *T. cruzi* | Brazil | TCC/USP:873 | TcII or TcIIb | *Homo sapiens* | 12 |
| FJ001629 | *T. cruzi* | Brazil | TCC/USP:1146 | TcII o TcIIb | *Homo sapiens* | 12 |
| FJ001631 | *T. cruzi* | Brazil | TCC/USP:417 | TcI | *Thyroptera tricolor* | 12 |
| FJ001632 | *T. cruzi* | Brazil | TCC/USP:507 | TcI | *Carollia perspicillata* | 12 |
| FJ001664 | *T. cruzi marinkellei* | Brazil | TCC/USP:344 |  | *Carollia perspicillata* (bat) | [[13]](#endnote-14) |
| FJ183394 | *T. cruzi* | Brazil | TryCC 45 | TcI | *Didelphis aurita* (opossum) | 13 |
| FJ549371 | *T. cruzi* | Brazil | TryCC125 | TcI | *Didelphis aurita* (opossum) | 13 |
| FJ549372 | *T. cruzi* | Brazil | TryCC128 | TcI | *Didelphis aurita* (opossum) | 13 |
| FJ549375 | *T. cruzi* | Brazil | TryCC363 and Roma 06 | TcI | *Didelphis marsupialis* (opossum) | 13 |
| FJ549377 | *T. cruzi* | Brazil | 884 | TcI | *Panstrongylus* | 13 |
| FJ549381 | *T. cruzi* | Brazisl | PAN ma 1 | TcI | *Philandeer frenata* | 13 |
| FJ549385 | *T. cruzi* | Paraguay | Arma13 | TcIII | *Dasypus novemcinctus* (armadillo) | 13 |
| FJ649484 | *T. cruzi marinkellei* | Australia |  |  | *Phascolarctos cinereus* (koala) | [[14]](#endnote-15) |
| FJ900239 | *T. cruzi* | Brazil | TryCC 417 |  | *Thyroptera tricolor* | 14 |
| FJ900240 | *T. cruzi* | Brazil | TryCC 507 |  | *Carollia perspicillata* | 14 |
| FJ900241 | *T. cruzi* | Brazil | TryCC 793 | TcBat | *Myotis levis* | 14 |
| JF746725 | *T. cruzi* | Colombia | AAA7 | TcII | ? | [[15]](#endnote-16) |
| JF746731 | *T. cruzi* | Colombia | Td3cl10 | TcII | ? | 15 |
| JN942610 | *T. cruzi* | Brazil | Y30 | TcII |  | [[16]](#endnote-17) |
| JN942611 | *T. cruzi* | Brazil | Y82 | TcII hybrid |  | 16 |
| JQ912642 | *T. cruzi* | Brazil | TC01 |  | Dog | Unpublished |
| JQ912643 | *T. cruzi* | Brazil | TC02 |  | Dog | Unpublished |
| JQ965535 | *T. cruzi* | Panama | 229_AJ_Guava | TcBat | *Artibeus jamaicensis* (bat) | [[17]](#endnote-18) |
| JQ965540 | *T. cruzi* | Panama | 141_AJ_Gigante | TcBat | *Artibeus jamaicensis* (bat) | 17 |
| JQ965541 | *T. cruzi* | Panama | 100_AJ_Guava | TcBat | *Artibeus jamaicensis* (bat) | 17 |
| JQ965543 | *T. cruzi* | Panama | 264_AJ_Leon | TcBat | *Artibeus jamaicensis* (bat) | 17 |
| JQ965544 | *T. cruzi* | Panama | 243_AJ_Guava | TcBat | *Artibeus jamaicensis* (bat) | 17 |
| JQ965547 | *T. cruzi* | Panama | 230_AJ_Guava | TcBat | *Artibeus jamaicensis* (bat) | 17 |
| JQ965548 | *T. cruzi* | Panama | 053AJGuanabano | TcBat | *Artibeus jamaicensis* (bat) | 17 |
| KF788250 | *T. cruzi* | Brazil | Mogi | TcI | *Panstrongylus megistus* (Triatominae) | [[18]](#endnote-19) |
| KR905433 | *T. cruzi* | Brazil | clone 1 |  | *Homo sapiens* (cardiac tissue) | 3 |
| KR905434 | *T. cruzi* | Brazil | clone 2 |  | *Homo sapiens* (cardiac tissue) | 3 |
| KR905435 | *T. cruzi* | Brazil | clone 3 |  | *Homo sapiens* (cardiac tissue) | 3 |
| KR905436 | *T. cruzi* | Brazil | clone |  | *Homo sapiens* (cardiac tissue) | 3 |
| KR905437 | *T. cruzi* | Brazil | clone | TcII or TcVI | *Homo sapiens* (cardiac tissue) | 3 |
| KR905438 | *T. cruzi* | Brazil | clone | TcI | *Homo sapiens* (cardiac tissue) | 3 |
| KR905439 | *T. cruzi* | Brazil | clone |  | *Homo sapiens* (cardiac tissue) | 3 |
| KT305894 | *T. cruzi* | Brazil | TCC 2557 | TcII | *Phyllostomus hastatus* | [[19]](#endnote-20) |
| KT305895 | *T. cruzi* | Brazil | TCC 2558 | TcII | *Myotis nigricans* (bat) | 19 |
| KT305896 | *T. cruzi* | Brazil | RNMO10 | TcI | *Peropteryx macrotis* (bat) | 19 |
| KT305897 | *T. cruzi* | Brazil | RNMO37 | TcI | *Molossus molossus* (bat) | 19 |
| KT305899 | *T. cruzi* | Brazil | APC1659 | TcI | *Platyrrhinus lineatus*(bat) | 19 |
| KT305900 | *T. cruzi* | Colombia | BatCha64 | TcI | *Micronycteris megalotis* (bat) | 19 |
| KT305901 | *T. cruzi* | Colombia | BatCha68 | TcI | *Myotis riparius* | 19 |
| KT305902 | *T. cruzi* | Colombia | BatCha70 | TcI | *Artibeus planirostris* | 19 |
| KT305907 | *T. cruzi* | Colombia | BatPra77 | TcBat | *Artibeus planirostris* | 19 |
| KT305909 | *T. cruzi* | Colombia | BatPra88 | TcBat | *Artibeus planirostris* | 19 |
| KT305913 | *T. cruzi* | Colombia | BatCha69 | TcBat | *Carollia perspicillata* (bat) | 19 |
| KT305914 | *T. cruzi* | Colombia | BatPra92 | TcBat | *Carollia perspicillata*(bat) | 19 |
| KT305915 | *T. cruzi* | Brazil | HMO117 | TcII | *Carollia perspicillata*(bat) | 19 |
| KT305916 | *T. cruzi* | Brazil | APC1640 | TcII | *Platyrrhinus lineatus* (bat) | 19 |
| KT305917 | *T. cruzi* | Venezuela | TCC 515 |  | *Homo sapiens* | 19 |
| KT305918 | *T. cruzi* | Brazil | TCC 669 |  | *Rhodnius robustus* (triatomine) | 19 |
| KT305919 | *T. cruzi* | Chile | Tulahuen cl2 |  | *Homo sapiens* | 19 |
| KT829462 | *T. cruzi* | Brazil | TCC873 | TcII + TcVI | *Homo sapiens* | [[20]](#endnote-21) |
| KX007998 | *T. cruzi* | China |  | TcI | *Homo sapiens* | Unpublished |
| KY748349 | *T. cruzi* | Brazil | 14CT |  | *Phyllostomus hastatus* | Unpublished |
| KY748353 | *T. cruzi* | Brazil | 276CT | TcII | *Artibeus jamaicensis* (bat) | Unpublished |
| KY748355 | *T. cruzi* | Brazil | 550CT |  | *Glossophaga soricina* (Bat) | Unpublished |
| KY748356 | *T. cruzi* | Brazil | 555CT |  | *Artibeus sp. (Bat)* | Unpublished |
| LT220258 | *T. cruzi* | Texas, USA | Gonzales 1 |  | *Triatoma gerstaeckeri* | [[21]](#endnote-22) |
| LT220259 | *T. cruzi* | Texas, USA | Gonzales 3 |  | *Triatoma gerstaeckeri* | 21 |
| LT220260 | *T. cruzi* | Texas, USA | Gonzales 5 |  | *Triatoma gerstaeckeri* | 21 |
| LT220261 | *T. cruzi* | Texas, USA | Gonzales 6 |  | *Triatoma gerstaeckeri* | 21 |
| LT220262 | *T. cruzi* | Texas, USA | Gonzales 7 |  | *Triatoma gerstaeckeri* | 21 |
| LT220263 | *T. cruzi* | Texas, USA | Gonzales 8 |  | *Triatoma gerstaeckeri* | 21 |
| LT220264 | *T. cruzi* | Texas, USA | Gonzales 9 |  | *Triatoma gerstaeckeri* | 21 |
| LT220265 | *T. cruzi* | Texas, USA | Gonzales 11 |  | *Triatoma gerstaeckeri* | 21 |
| LT220266 | *T. cruzi* | Texas, USA | Gonzales 12 |  | *Triatoma gerstaecker* | 21 |
| LT220267 | *T. cruzi* | Texas, USA | Gonzales 15 |  | *Triatoma gerstaeckeri* | 21 |
| LT220268 | *T. cruzi* | Texas, USA | ITRI/MX/99/Cari-006 |  | *rodent* | 21 |
| LT220273 | *T. cruzi* | Texas, USA | San Marcos II-2 |  | *Triatoma gerstaeckeri* | 21 |
| LT220279 | *T. cruzi* | Texas, USA | Las Palomas 196 |  | *Baiomys taylori* | 21 |
| LT220280 | *T. cruzi* | Texas, USA | Las Palomas 435 |  | *Liomys irroratus*(mexican muse) | 21 |
| LT220281 | *T. cruzi* | Texas, USA | Las Palomas 628 |  | *Peromyscus leucopus (mouse)* | 21 |
| M31432 | *T. cruzi* | Mexico |  |  |  | [[22]](#endnote-23) |
| MF141875 | *T. cruzi* | Brazil | LBT 4874 | TcII/TcVI |  | [[23]](#endnote-24) |
| MF141882 | *T. cruzi* | Brazil | LBT 5075 | TcII/TcVI |  | 23 |
| MF141885 | *T. cruzi* | Brazil | LBT 5078 | TcII/TcVI |  | 23 |
| MF141889 | *T. cruzi* | Brazil | LBT 5791 | TcII/TcVI |  | 23 |
| MH047278 | *T. cruzi* | Mexico | MDID/MX/1991/VER003 |  | *Didelphis sp.* | Unpublished |
| MH047279 | *T. cruzi* | Mexico | MDID/MX/1991/VER006 |  | *Didelphis sp.* | Unpublished |
| MH059789 | *T. cruzi* | Brazil | SC96 cl3 | TcII | *Homo sapiens* | 2 |
| MH059790 | *T. cruzi* | Brazil | SI8 cl1 | TcII | *Triatoma sordida* | 2 |
| MH059791 | *T. cruzi* | Brazil | SI7 | TcII | *Triatoma sordida* | 2 |
| MH059792 | *T. cruzi* | Brazil | FAMEMA | TcII | *Homo sapiens* | 2 |
| MH059793 | *T. cruzi* | Brazil | SIGR3 cl1 | TcII | *Felis catus* | 2 |
| MH411621 | *T. cruzi* | Brazil | bPgy 89 |  | *Lycalopex gymnocercus* | [[24]](#endnote-25) |
| MH411622 | *T. cruzi* | Brazil | bPgy 90 |  | *Lycalopex gymnocercus* | 24 |
| MH411623 | *T. cruzi* | Brazil | CPAGM 796 | TcI | *Cerdocyon thous* | 24 |
| MH411624 | *T. cruzi* | Brazil |  | TcI | *Cerdocyon thous* | 24 |
| MH411625 | *T. cruzi* | Brazil | CPAPGM 850 | TcI | *Cerdocyon thous* | 24 |
| MH411626 | *T. cruzi* | Brazil | CPAPGM 855 | TcI | *Cerdocyon thous* | 24 |
| MH411627 | *T. cruzi* | Brazil | LBT 6867 |  | *Artibeus lituratus* | 24 |
| MH411628 | *T. cruzi* | Brazil | LBT 6895 |  | *Phyllostomus hastatus* | 24 |
| MH411629 | *T. cruzi* | Brazil |  | TcII | *Artibeus fimbriatus* | 24 |
| MH411630 | *T. cruzi* | Brazil |  |  | *Marmosa sp* | 24 |
| MH411631 | *T. cruzi* | Brazil | PCE 408 |  | *Didelphis albiventris* | 24 |
| MH411632 | *T. cruzi* | Brazil | PCE 416 |  | *Didelphis albiventris* | 24 |
| MH411634 | *T. cruzi* | Brazil | LBCE 19674 | TcII | *Philander sp* | 24 |
| MH411635 | *T. cruzi* | Brazil | LBCE 19675 |  | *Didelphis marsupialis* | 24 |
| MH411636 | *T. cruzi* | Brazil | LBCE 19687 |  | *Philander sp* | 24 |
| MH411637 | *T. cruzi* | Brazil |  |  |  | 24 |
| MH411638 | *T. cruzi* | Brazil | LBCE 19689 |  | *Didelphis marsupialis* | 24 |
| MH411639 | *T. cruzi* | Brazil | YL 865 | TcI | *Monodelphis americana* | 24 |
| MH411640 | *T. cruzi* | Brazil | LBCE 18616 |  | *Gracilinanus agilis* | 24 |
| MH411644 | *T. cruzi* | Brazil | EM 715 | TcI | *Glossophaga soricina* | 24 |
| MH411685 | *T. lainsoni* | Brazil | LBCE 18652 clone 3 |  | *Gracilinanus agilis* | 24 |
| MH538953 | *T. cruzi* | Costa Rica | TcruziOpossum6CR |  | *Philander*(opossum) | Unpublished |
| MH538954 | *T. cruzi* | Costa Rica | TcruziOpossum7CR |  | *Philander*(opossum) | Unpublished |
| MH538955 | *T. cruzi* | Costa Rica | TcruziRaccoon1CR |  | *Procyon lotor* | Unpublished |
| MH538956 | *T. cruzi* | Costa Rica | TcruziRaccoon2CR |  | *Procyon spp.* | Unpublished |
| MH538957 | *T. cruzi* | Costa Rica | TcruziRaccoon10CR |  | *Procyon lotor* | Unpublished |
| MH538958 | *T.cruzi* | Costa Rica | TcruziRaccoon5CR |  | *Procyon spp.* | Unpublished |
| MH538959 | *T. cruzi* | Costa Rica | TcruziRaccoon7CR |  | *Procyon spp.* | Unpublished |
| MH538960 | *T. cruzi* | Costa Rica | TcruziRaccoon9CR |  | *Procyon spp.* | Unpublished |
| MH538961 | *T, cruzi* | Costa Rica | TcruziRaccoon3CR |  | Procyon spp | Unpublished |
| MH538962 | *T. cruzi* | Costa Rica | TcruziRaccoon6CR |  | Procyon spp | Unpublished |
| MH538963 | *T. cruzi* | Costa Rica | TcruziRaccoon4CR |  | Procyon spp | Unpublished |
| MH538964 | *T. cruzi* | Costa Rica | TcruziRaccoon8CR |  | *Procyon spp* | Unpublished |
| MK640442 | *T. cruzi* | Mexico (try18F) |  | TcII | *Hommo sapiens* | This work |
| X53917 | *T. cruzi* | Peru | Peru | TcII or TcIIb |  | [[25]](#endnote-26) |

In color are highlighting the case sequence and other sequences presented in the same haplotype that correspond to DTU TcII.

**References**

1. Kawashita SY, Sanson GF, Fernandes O, et al, 2001. Maximum-likelihood divergence date estimates based on rRNA gene sequences suggest two scenarios of Trypanosoma cruzi intraspecific evolution. Mol. Biol. Evol.;18 (12): 2250-2259. <https://doi.org/10.1093/oxfordjournals.molbev.a003771> [↑](#endnote-ref-2)
2. Ribeiro AR, Lima L, de Almeida LA, Monteiro J, Moreno CJ, Nascimento JD, de Araujo RF, Mello F, Martins LP, Graminha MA, Teixeira MM, Silva MS, Steindel M, da Rosa JA. Biological and Molecular Characterization of *Trypanosoma cruzi* Strains from Four States of Brazil. Am J Trop Med Hyg. 2018;98(2):453-463 [↑](#endnote-ref-3)
3. DarioMA, RodriguesMS, BarrosJH, XavierSC, D'AndreaPS, RoqueAL, JansenAM. Ecological scenario and Trypanosoma cruzi DTU characterization of a fatal acute Chagas disease case transmitted orally (Espirito Santo state, Brazil). Parasit Vectors 2016;9: 477<https://doi.org/10.1186/s13071-016-1754-4>. [↑](#endnote-ref-4)
4. Sanson GFO, Kawashita S, Brunstein A, et al, 2002. Experimental Phylogeny of neutrally evolvingDNA sequences generated by a bifurcate series of nested polymerase chain reactions. Mol Biol Evol 19(2):170-178.<https://doi.org/10.1093/oxfordjournals.molbev.a004069> [↑](#endnote-ref-5)
5. Stevens J, Noyes H, Gibson W, 1998. The evolution of *Trypanosomes* Infecting humans and primates. Men Inst Oswaldo Cruz 93)5=:669-676. [↑](#endnote-ref-6)
6. Stevens JR, Noyes HA, Dover GA et al, 1999. The ancient and divergent origins of the human pathogenic trypanosomes, *Trypanosoma brucei* and *T. cruzi*. Parasitology 118:107-116. <https://doi.org/10.1186/s13071-015-1255-x> [↑](#endnote-ref-7)
7. Dasilva M, Noyes H, Campaner M et al, 2004. Phylogenytaxonomy and grouping of *Trypanosoma rangeli* isolates from man, triatomines and sylvatic mammals from widespread geographical origin based on SSU and ITS ribosomal sequences. Parasitology129, 549–561.<https://doi.org/10.1017/S0031182004005931> [↑](#endnote-ref-8)
8. Franzé O, Talavera-López C, Ochanya S et al, 2012. Comparatiive genomic análisis of human infective Trypanosoma cruzi lineages with the bat-restricted subspecies T. cruzi marinkellei. BMC Genomics13:531. <https://doi.org/10.1186/1471-2164-13-531> [↑](#endnote-ref-9)
9. Franzén O1, Ochaya S, Sherwood E, et al, 2011. Shotgun sequencing analysis of Trypanosoma cruzi I Sylvio X10/1 and comparison with T. cruzi VI CL Brener. PLoS Negl Trop Dis. 8;5(3):e984. <https://doi.org/10.1371/journal.pntd.0000984> [↑](#endnote-ref-10)
10. Marcili A, Valente V, Valente S. et al, 2009.Trypanosoma cruziin Brazilian Amazonia: Lineages TCI and TCIIa in wild primates, Rhodniusspp. and in humans with Chagas disease associated with oral transmisión. Int J Parasit 39 615–623. <https://doi.org/10.1016/j.ijpara.2008.09.015> [↑](#endnote-ref-11)
11. da Silva, MarciliA, Lima L et al, 2009. Trypanosoma rangeliisolates of bats from Central Brazil: Genotyping and phylogenetic analysis enable description of a new lineage using spliced-leader gene sequences. Acta Tropica 109: 199–207. <https://doi.org/10.1016/j.actatropica.2008.11.005> [↑](#endnote-ref-12)
12. Marcili A, Lima L, Cavazzana M, et al, 2009. A new genotype of Trypanosoma cruzi associated with bats evidenced by phylogenetic analyses using SSU rDNA, cytochrome b and Histone H2B genes and genotyping based on ITS1 rDNA. Parasitology, 136(6), 641-655. <https://doi.org/10.1017/S0031182009005861> [↑](#endnote-ref-13)
13. Cavazzana M Jr, Marcili A, Lima L et al, 2010. Phylogeographical, ecological and biological patterns shown by nuclear (ssrRNA and gGAPDH) and mitochondrial (Cyt b) genes of trypanosomes of the subgenus Schizotrypanum parasitic in Brazilian bats. Int J Parasitol 1;40(3):345-55. <https://doi.org/10.1016/j.ijpara.2009.08.015> [↑](#endnote-ref-14)
14. McInnes L., Gillett A, Ryan U., et al, 2009. Trypanosoma irwini n. sp (Sarcomastigophora: Trypanosomatidae) from the koala (Phascolarctos cinereus). Parasitology, 136(8), 875-885. <https://doi.org/10.1017/S0031182009006313> [↑](#endnote-ref-15)
15. Ramírez JD,Duque MC,Montilla M, et al 2012. Natural and emergent *Trypanosoma cruzi* I genotypes revealed by mitochondrial (Cytb) and nuclear (SSU rDNA) genetic markers. Exp Parasitol 132(4): 487-494. <https://doi.org/10.1016/j.exppara.2012.09.017> [↑](#endnote-ref-16)
16. Cortez C, Martins RM, Alves RM, Silva RC, Bilches LC, et al, 2012. Differential Infectivity by the Oral Route of *Trypanosoma cruzi* Lineages Derived from Y Strain. PLoS Negl Trop Dis 6(10): e1804. <https://doi.org/10.1371/journal.pntd.0001804> [↑](#endnote-ref-17)
17. Pinto CM, Kalko EK, Cottontail I, Wellinghausen N, Cottontail VM. TcBat a bat-exclusive lineage of *Trypanosoma cruzi* in the Panama Canal Zone, with comments on its classification and the use of the 18S rRNA gene for lineage identification. Infect Genet Evol 2012;12 (6):1328-1332 [↑](#endnote-ref-18)
18. Martins LPA, Castanho REP, Therezo ALS, Ribeiro AR, Lima L, Teixeira MMG, Sperança MA, Rodrigues VLC, da Rosa JA. Biological and molecular characterization of a *Trypanosoma cruzi* isolate obtained from Panstrongylus megistus captured in Sao Paulo State, Brazil. Acta Parasitologica 2015;60(1):65-74.<https://doi.org/10.1515/ap-2015-0009> [↑](#endnote-ref-19)
19. Lima,L, Espinosa-AlvarezO, OrtizPA, Trejo-VaronJA,Carranza,JC, Pinto,CM, BuckGA, CamargoEP, Teixeira,MM. Genetic diversity of Trypanosoma cruzi in bats, and multilocus phylogenetic and phylogeographical analyses supporting Tcbat as an independent DTU (discrete typing unit). Acta Trop 2015;151:166-77. <https://doi.org/10.1016/j.actatropica.2015.07.015>. [↑](#endnote-ref-20)
20. PintoCM, Ocana-MayorgaS, TapiaEE, LobosSE, ZuritaAP,Aguirre-VillacisF, MacDonaldA, VillacisAG, LimaL,TeixeiraMM, GrijalvaMJ, PerkinsSL.Bats, Trypanosomes, and Triatomines in Ecuador: New Insights into the Diversity, Transmission, and Origins of Trypanosoma cruzi and Chagas Disease. PLoS ONE 2015;10(10): E0139999 <https://doi.org/10.1371/journal.pone.0139999>. [↑](#endnote-ref-21)
21. Aleman A, Guerra T, Maikis TJ, Milholland MT, Castro-Arellano I, Forstner MR, Hahn D. The Prevalence of Trypanosoma cruzi, Causal Agent of Chagas Disease, in Texas Rodent Populations. Ecohealth. 2017;14(1):130-143. <https://doi.org/10.1007/s10393-017-1205-5>. [↑](#endnote-ref-22)
22. Hernandez R, Rios P, Valdes AM, Pinero D. Primary structure of *Trypanosoma cruzi* small-subunit ribosomal RNA coding region: comparison with other trypanosomatids. Mol. Biochem. Parasitol. 1990;41(2):207-212. [↑](#endnote-ref-23)
23. Dario MA, Lisboa CV, Costa LM, Moratelli R, Nascimento MP, Costa LP, Leite YL., Llewellyn MS, Xavier SC, Roque AL, Jansen AM. High *Trypanosoma* spp. diversity is maintained by bats and triatomines in Espírito Santo state, Brazil. PLoS One. 2017;12(11):e0188412. <https://doi.org/10.1371/journal.pone.0188412> [↑](#endnote-ref-24)
24. RodriguesMS, LimaL, XavierSC, HerreraHM, RochaFL,RoqueAL, TeixeiraMM, JansenAM. Uncovering Trypanosoma spp. diversity of wild mammals by the use of DNA from blood clots. Int J Parasitol Parasites Wildl 2019;8:171-181. <https://doi.org/10.1016/j.ijppaw.2019.02.004>. [↑](#endnote-ref-25)
25. Fernandes AP, Nelson K, Beverley SM. Evolution of nuclear ribosomal RNAs in kinetoplastid protozoa: perspectives on the age and origins of parasitism. Proc. Natl. Acad. Sci. U.S.A. 1993;90(24):11608-11612. [↑](#endnote-ref-26)
