# Supplementary figures and images for: Trypanosoma cruzi infection associated with atypical clinical manifestation during the acute phase of the Chagas disease
Source: Parasit Vectors. 2019 Oct 30;12:506. doi: 10.1186/s13071-019-3766-3 (PMC6822409; doi:10.1186/s13071-019-3766-3)

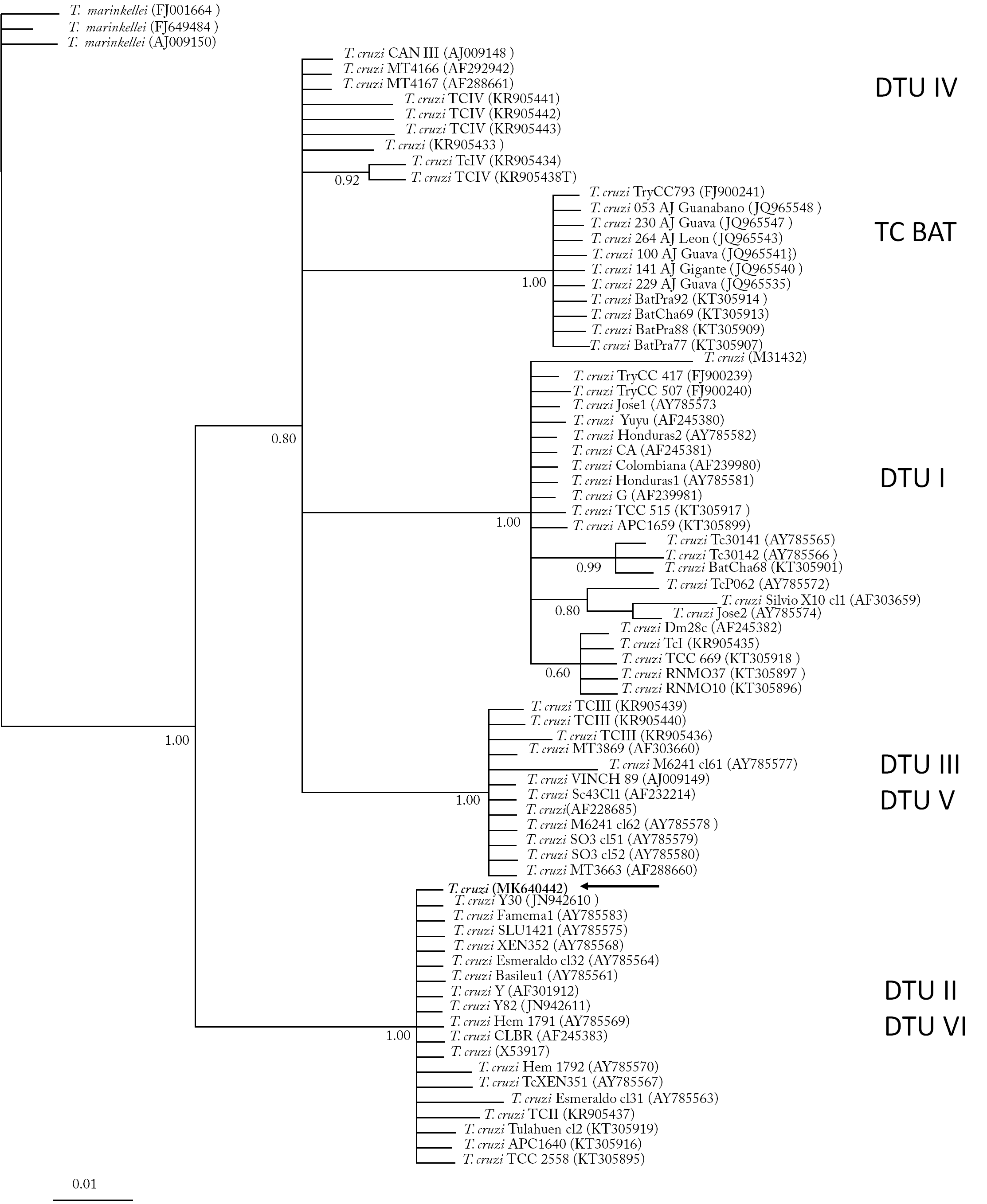

Supplement: Supplementary file 2 — Additional file 2: Figure S1. Phylogenetic reconstruction using SSU rDNA for different trypanosomatid species available on GenBank. [file 13071_2019_3766_MOESM2_ESM.tif]
