# Supplementary material for: Trypanosoma cruzi infection associated with atypical clinical manifestation during the acute phase of the Chagas disease
Source: Parasit Vectors. 2019 Oct 30;12:506. doi: 10.1186/s13071-019-3766-3 (PMC6822409; doi:10.1186/s13071-019-3766-3)
Supplement: Supplementary file 3 — Additional file 3: Text S1. Sequence obtained from the patient for the satelite-DNA region of Trypanosma cruzi. [file 13071_2019_3766_MOESM3_ESM.doc]

**Additional file 3: Text S1.** Sequence obtained from the patient for the satelite-DNA region of *Trypanosma cruzi.*

AGCTCTTGCCCACACGGGTGCTGCAGTCGGCGGATCGTTTTCGAGCGGCTGCTGCACCACACGTTGTGGTCTAAGTTTTTGTTTCGAATTATGAATGGCGGGAGTCAGAGGCACTCTCTTTCAATGTATGTTTGCGTGTGCACACACTGGACACCAAACAACCCTGAACTATCCGCTGCTTGG
